# Epidemiology and management of hepatitis C virus infections in immigrant populations

**DOI:** 10.1186/s40249-019-0528-6

**Published:** 2019-03-15

**Authors:** Nicola Coppola, Loredana Alessio, Lorenzo Onorato, Caterina Sagnelli, Margherita Macera, Evangelista Sagnelli, Mariantonietta Pisaturo

**Affiliations:** 1 Department of Mental Health and Public Medicine, Section of Infectious Diseases, University of Campania, Via: L. Armanni 5, 80131 Naples, Italy; 2 Infectious Diseases Unit, AORN Sant’Anna e San Sebastiano di Caserta, 81100 Caserta, Italy

**Keywords:** HCV infection, Chronic hepatitis C, immigrant, Directly acting antiviral agent, Low-income country

## Abstract

**Background:**

At present, there is a continuous flow of immigrants from the south of the world to north-western countries. Often immigrants originate from areas of high-prevalence of viral hepatitis and pose a challenge to the healthcare systems of the host nations. Aims of this study is to evaluate the prevalence and virological and clinical characteristics of hepatitis C virus (HCV) infection in immigrants and the strategies to identify and take care of the immigrants infected with HCV.

**Main body:**

We conducted an electronic literature search in several biomedical databases, including PubMed, Google Scholar, Scopus, Web of Science, using different combinations of key words: “HCV infection; chronic hepatitis C, immigrants; low-income countries”. We included studies written in English indicating the epidemiological data of HCV infection in the immigrant population, studies that assessed the clinical presentation, clinical management and treatment with directly acting antiviral agent in immigrants,

HCV infection is unevenly distributed in different countries, with worldwide prevalence in the general population ranging from 0.5 to 6.5%. In Western countries and Australia this rate ranges from 0.5 to 1.5%, and reaches 2.3% in countries of south-east Asia and eastern Mediterranean regions, 3.2% in China, 0.9% in India, 2.2% in Indonesia and 6.5% in Pakistan; in sub-Saharan Africa the prevalence of HCV infection varies from 4 to 9%. Immigrants and refugees from intermediate/high HCV endemic countries to less- or non-endemic areas are more likely to have an increased risk of HCV infection due to HCV exposure in their countries of origin. Because of the high HCV endemicity in immigrant populations and of the high efficacy of directly acting antiviral agent therapy, a campaign could be undertaken to eradicate the infection in this setting.

**Conclusions:**

The healthcare authorities should support screening programs for immigrants, performed with the help of cultural mediators and including educational aspects to break down the barriers limiting access to treatments, which obtain the HCV clearance in 95% of cases and frequently prevent the development of liver cirrhosis and hepatocellular carcinoma.

**Electronic supplementary material:**

The online version of this article (10.1186/s40249-019-0528-6) contains supplementary material, which is available to authorized users.

## Multilingual abstracts

Please see Additional file 1 for translations of the abstract into the five official working languages of the United Nations.

## Background

Since ancient times, the history of humanity has been greatly influenced by numerous migratory phenomena that have determined the current ethnic composition of nations. At present, there is a continuous flow of immigrants from the south of the world to north-western countries, due to the inability to achieve economic and social development in geographical areas where dictatorial regimens have been responsible for wars between states, civil wars and tribal clashes that have impoverished the population and sown hatred and famine. The immigrants are motivated to leave their native countries for a variety of reasons, including political issues, escaping conflict or natural disaster, a desire for economic prosperity or family re-unification.

Often immigrants originate from areas of high-prevalence of viral hepatitis and pose a challenge to the healthcare systems of the host nations. The lack of universal standards for screening, vaccination and treatment of viral hepatitis causes a continuous increase in the burden of chronic liver disease among immigrant populations globally.

Studies have identified patient factors such as lack of knowledge, late presentation and poor adherence to follow-up and fear of the side effects of treatment as major barriers for effective treatment of viral hepatitis [1, 2]. The identification of barriers to treatment is critical since studies show that improving sustained virological response (SVR) rates is not enough to improve hepatitis C virus (HCV) eradication rates without a concurrent increase in treatment uptake [3]. Therefore, even if the cost-effectiveness of universal screening of immigrants for HCV has not been clearly established, since HCV prevalence varies widely among host nations and immigrant groups and is likely to depend on the expected HCV prevalence, treatment uptake rates, and expected SVR [4], efforts to increase case identification and treatment among immigrants should be made.

In this review the authors try to outline the management of immigrants with chronic HCV infection, one of the most frequent and serious health problems both in immigrant and host populations. Today, new treatments with directly acting antiviral agents (DAAs), expensive drugs of good tolerability, obtain HCV clearance in 95% of cases and frequently prevent the development of liver cirrhosis and hepatocellular carcinoma (HCC). So, in this study the authors evaluated the prevalence and virological and clinical characteristics of hepatitis C virus (HCV) infection in the immigrant population and the strategies to identify and take care of the immigrants infected with HCV; the immigrants were defined as subjects from endemic areas for HCV infection, mostly the south of the world, to less- or non-endemic areas, mostly north-western countries. In the DAA era it is important to recognize the immigrants with HCV infection to eradicate it.

## Search strategy

We conducted an electronic literature search in several biomedical databases, including PubMed, Google Scholar, Scopus, Web of Science, using different combinations of key words: “HCV infection; chronic hepatitis C, immigrants; low-income countries”.

We included studies written in English indicating the epidemiological data of HCV infection in the immigrant population, studies that assessed the clinical presentation, clinical management and treatment with DAAs in immigrants, excluding those that presented the data only in autochthonous patients of host nations and those not written in the English language.

Among the 569 potentially relevant articles identified from the search of electronic databases, we found the studies included in the text and in the tables.

## Main text

### Global trend in immigration

According to the 2017 data by the United Nations Department of Economic and Social Affairs (UN DESA) report, 258 million people are living out of their country of origin, with a 49% increase in the last 15 years [5], a figure not including the undocumented immigrants who account for 10–15% of the immigration flow worldwide [6]. In 2017, 74% of immigrants were of working age (between 20 and 64 years of age), a percentage higher than that of the global population (57%), and women comprise 48.4% of all immigrants [5, 7]. Most immigrants move from the south of the world to north and western countries, especially Europe and North America.

In 2016, the immigrants living in the 28 countries of the European Union were 37 million, accounting for 7.3% of the total population, mainly distributed in 5 countries: Germany (23.4%), United Kingdom (15.3%), Italy (13.6%), Spain (12%) and France (11.9%) [8].

The countries of origin of the immigrants have a high or intermediate endemicity for several parenteral infections, such as human immunodeficiency virus (HIV), HCV and hepatitis B virus (HBV) infection; instead, the hosting countries have a low or no endemicity for these infections. The immigrant population meets several obstacles in accessing the essential healthcare services in the host country, including cultural and language barriers, lack of inclusive health policies for immigrants and the illegal status for undocumented immigrants. These obstacles have a disruptive impact on the well-being of the immigrant population since they reduce the possibility of obtaining satisfactory results in terms of public health, such as the control and prevention of HIV, HCV, HBV infection, tuberculosis, malaria and human influenza. The high morbidity and mortality rates of these infections raise critical health concerns that deserve attention by the Healthcare Authorities.

## HCV infection

### Virological and epidemiological characteristics

Hepatitis C virus is a hepatotropic member of the Flaviridae family. Seven main genotypes (with 30–35% variation at the nucleotide level) and 67 subtypes (with less than 15% difference at the nucleotide level) have so far been described, each composed of a myriad of viral quasi-species [9–12]. Discovered in 1989, HCV has been considered responsible for more than half of the cases of chronic liver disease worldwide [13].

Exposure to infected blood or blood products (intravenous drug use, iatrogenic exposure, tattooing, piercing) and risky sexual habits (multiple partners, anal sex, presence of genital lesions) are the risk factors most frequently associated to HCV transmission [14–18]. Perinatal transmission from HCV-infected mothers to their newborn babies has a limited epidemiologic role since it occurs in less than 5% of the cases, a prevalence increased in the case of HCV/HIV coinfection [19]. Needlestick is a frequent event in health care workers, but its epidemiologic role is limited most probably because of the low quantity of blood usually carried by contaminated needles [20].

### Global epidemiology of HCV infection

The WHO estimated that 71 million people were living with HCV infection in 2015, accounting for 1% of the world population. The infection is unevenly distributed in different countries, with a prevalence in the general population ranging from 0.5 to 6.5%. In Western countries and Australia this rate ranges from 0.5 to 1.5%; it reaches 2.3% in countries of south-east Asia and in eastern Mediterranean regions [21], 3.2% in China, 0.9% in India, 2.2% in Indonesia and 6.5% in Pakistan [22].

The global distribution of each HCV genotype varies in different geographical regions. HCV genotype 1 is the most prevalent worldwide and has a widespread geographical distribution, representing 46% of all HCV infections. HCV genotype 3 is the second most prevalent genotype and accounts for 30% of global infections, and is more common in south Asia, Australia, and in some countries in Europe. HCV genotypes 2 and 4 represent 9–13% of HCV infections with a more limited geographical distribution; HCV genotype 2 prevalence is higher in Asia and West Africa, while a high incidence of HCV genotype 4 infection occurs in central and eastern sub-Saharan Africa, North Africa, and the Middle East. HCV genotypes 5, 6, and 7 are the most restricted in geographical distribution, with genotype 5 common in South Africa and genotype 6 prevalent in east and south-east Asia, while genotype 7 infection has been reported in a small number of individuals from the Democratic Republic of Congo [10, 11].

### Clinical aspects

After HCV infection has been acquired, nearly 35% of subjects clear the virus spontaneously or after a self-limited asymptomatic acute hepatitis, whereas the remaining 65% progress to chronicity, usually identified by the persistence of HCV RNA in the serum after 6 months [23].

The natural history of chronic HCV infection is extremely variable, ranging from a long-term persistence of minimal liver changes with an indolent progression of liver fibrosis to a rapid transition to liver cirrhosis and the development of severe complications such as portal hypertension, liver failure and HCC [24, 25]. In most cases, however, liver fibrosis shows a slow progression and liver cirrhosis develops in about 20% of patients within 20 years [24, 25]. Several factors may speed the progression of liver disease, including old age at the time of infection, concomitant alcohol abuse, a presence of diabetes or other metabolic disorders, an impaired host immune response of different nature, including coinfection with HIV and coinfection with HBV [23–28].

### Therapeutic management

The combination of pegylated interferon-alpha (peg-IFN) plus ribavirin (RBV) was the treatment of choice for HCV-related chronic infection for more than a decade, providing a sustained HCV clearance in half of the patients infected with HCV-genotype 1, in around 75% of those with HCV-genotype 2 and in around 60% of those with HCV-genotype 3. However, a non-satisfactory therapeutic response and the high frequency and severity of side effects of interferon-based treatments have urged pharmaceutical companies to develop more effective and better-tolerated drugs [29–35], termed directly acting antivirals (DAAs), which have revolutionized the treatment of HCV infection. Interferon-free treatment with DAAs provides excellent chances for a sustained HCV elimination and can prevent the progression of the liver disease. According to guidelines there are different treatment schedules, depending on the HCV genotype and sub-type and on the presence or absence of cirrhosis (Table 1). These new oral interferon-free DAA regimens provide an SVR in nearly 95% of patients treated [34, 35].

**Table 1:** directly acting antiviral agent (DAA) regimens and specific indication according to HCV genotype and severity of liver disease

DAA regimen	HCV regions covered	HCV Genotype	Pill/day	Duration in weeks
Ombitasvir/paritaprevir/ritonavir/dasabuvir	NS3 + NS5A + NS5B	1,4	4	
- Non cirrhosis				8^a^
- Cirrhosis				12
Elbasvir/grazoprevir	NS3 + NS5A	1,4	1	12^b^
Sofosbuvir/velpatasvir	NS5A + NS5B	1-6	1	12
Glecaprevir/pibrentasvir	NS3 + NS5A	1-6	3	
- Non cirrhosis				8
- Cirrhosis				12

^a^ genotype 1b; *NS3* non-structural 3 region, *NS5A* non-structural protein 5A, *NS5B* non-structural protein 5B

^b^16 weeks for GT1a with high viral load and/or NS5A RASs

## HCV infection in immigrant populations

### Risk factors for HCV infection in immigrant populations

Immigrants from the south of the world are at a particularly high risk for HCV infection because they come from countries with a high or intermediate endemicity; moreover, they have scanty knowledge of this infectious disease and of the routes of transmission due to the lack of access to healthcare and to the difficulty in obtaining healthcare information from the media. Other important points to evaluate are that they are mostly young, sexually active and have been subjected to tribal rituals that may favor the parenteral spread of this infection.

Whereas the improvement in blood screening and in the sterilization of medical devices has produced a decline in HCV transmission in western Europe and North America, where HCV is currently transmitted mostly through the injection of illicit drugs [36], iatrogenic exposure and tribal rituals remain the major route of transmission in developing countries [37].

A retrospective cohort study [38] conducted in Canada from 1998 to 2007 compared 1821 immigrants and 18 318 non-immigrants, all HCV-positive and hospitalized. Compared with immigrants, non-immigrants showed a higher proportion of males and a higher rate of intravenous drug users and HCV/HIV coinfection. A US study group compared the prevalence and correlates of alcohol abuse among US foreign-born versus US-natives by race-ethnicity, experiencing that the foreign-born had lower rates of alcohol abuse than the US-born, but some variations were noted by race-ethnicity. The risk of alcohol abuse could be due to stressful and traumatic experiences of war, torture, and potentially traumatic migration journeys [39–41].

The Greenaway group [42] reported that HCV immigrants presented a mean delay in diagnosis and linkage to care of almost 10 years after arrival, which could explain, together with a high rate of HIV coinfection, the higher incidence of HCC in this group.

### Prevalence of HCV infection in immigrant populations

The prevalence of HCV infection in the immigrant populations varies according to the countries of origin. Table 2 shows data of HCV seroprevalence, comparing in each country/region the prevalence of HCV in the general population vs immigrant population [43–52].

**Table 2:** Prevalence of HCV infection in immigrant populations according to the area of origin and prevalence of HCV infection in the indigenous population

Author, year	Country/ HCV-Ab prevalence in general population (ECDC) [43]	Number of patients	Enrolment period	Area of origin *n* (%)	HCV-Ab- positive *n* (%)	HCV-RNA- positive *n* (%)
Tafuri, 2010 [50]	Italy 2.1–8%	529	May–July 2008	Africa: 510 (96.4)	Total: 24 (4.5)	Not reported
				Asia 19 (3.6)		
Bottecchia, 2011 [45]	Spain 1.1–2%	1718	January 2007– December 2008	Africa: 1322 (77); Latin America: 378 (22)	Total: 212 (12.3)	Not reported
				Asia: 18 (1)		
Urbanus, 2011 [47]	Netherlands < 0.5%	5580	2003–2009	Western countries: 720 (22.9)	2 (0.3)	2 (0.3)
				Non-western countries: 4860 (87.1)	34 (0.7)	28 (0.6)
Rivas, 2013 [46]	Spain 1.7%	1493	January 2002– December 2008	Equatorial Guinea: 1220 (81.6)	234 (19.2)	141 (11.6)
				Other sub-Saharan countries: 276 (18.4)	2 (0.7)	0 (0.0)
Stornaiuolo, 2013 [51]	Italy 2.1–8%	2681	1999–2009	North Africa: 101 (3.8%)	10 (9.9)	Not reported
				Sub-Saharan Africa: 2202 (82.3%)	54 (2.5)	
				Eastern Europe: 211 (7.9%)	15 (7.1)	
				Asia: 115 (4.3%)	4 (3.5)	
Coppola, 2013 [48]	Italy 2.1–8%	882	January 2012– June 2013	Northern Africa: 80 (9.1)	2 (2.5)	Not reported
				Sub-Saharan Africa: 444 (50.3)	17 (3.8)	
				Eastern Europe: 198 (22.5)	12 (6.1)	
				India-Pakistan area: 126 (14.3)	9 (7.1)	
Daw, 2016 [44]	Libya 1.2%	14 205	2013–2015	Central Africa: 2557 (18.0)	146 (5.7)	Not reported
				West Africa: 4993 (35.1)	405 (8.1)	
				Horn of Africa: 3524 (24.8)	296 (8.4)	
				North Africa: 3131 (22.1)	314 (10.0)	
Sagnelli, 2018 [49]	Italy 2.1–8%	1727	2012–2017	Northern Africa: 94 (5.4)	2 (2.2)	1 (1.1)
				Sub-Saharan Africa: 1084 (62.8)	35 (3.6)	9 (0.9)
				Eastern Europe: 261 (15)	15 (5.7)	11 (4.2)
				India-Pakistan area: 371 (21.5)	16 (4.3)	9 (2.4)
				Other countries: 29 (1.7)	2 (6.9)	1 (3.4)
Jablonka, 2017 [52]	Germany < 0.5%	604	August–September 2015	European Region: 41 (6.8)	5 (1.0)	Total: 4 (0.6)
				African Region: 55 (9.1)	1 (2.4)	
				Eastern Mediterranean Region: 482 (79.8)	1 (1.8)	
				South East Asia: 7 (1.2)	0 (0.0)	
				Unknown: 19 (3.1)	0 (0.0)	

*ECDC* European center for diseases prevention and control

Bottecchia et al. [45] screened for HCV, HBV and HIV 1718 consecutive immigrants referring to a Spanish institution in 2007–2008, of whom 77% were from sub-Saharan Africa, and found a seroprevalence of 12.3, 7 and 7.9%, respectively. The same authors evaluated the prevalence of HCV infection in a cohort of 1220 subjects from Equatorial Guinea living in Spain in 2002–2008 and detected anti-HCV positivity in 19.2% and HCV-RNA positivity in 11.6% of cases [46]. In 2011, Urbanus and colleagues [47] reported the data of four HCV seroprevalence surveys enrolling either heterosexual patients referring to the sexually-transmitted-infections clinics in Amsterdam, randomly selected pregnant women, a randomly selected sample of inhabitants in Amsterdam or a randomly selected sample of inhabitants of the Netherlands, for a total of 4860 non-Western and 9329 Western participants. Worthy of note, the HCV seroprevalence ranged from 0.7 to 2.3% in non-Western first-generation immigrants from different countries and from 0 to 1.6% in the second-generation of non-Western immigrants. Our research group found a 4% anti-HCV seroprevalence in 882 undocumented immigrants/low-income refugees from different countries and living in the urban areas of Naples and Caserta in southern Italy in 2012–2013 [48], and, more precisely, in 2.5% of immigrants from north Africa, in 6.1% of those from Eastern Europe and in 7.1% of those from the India-Pakistan subcontinent. Updated results of this study regarded 1727 immigrants screened from 2012 to 2015 [49], of whom 70 (4.1%) were anti-HCV-positive. Of these 70, 31 (44.3%) were HCV-RNA-positive with a mean viral load of 2.2 × 10^7^. This anti-HCV prevalence is similar to those reported by other authors in southern Italy: 4.5% by Tafuri et al. [50] in 529 refugees observed in 2008 in the Apulia region and 3.6% found by Stornaiuolo et al. [51] in 2681 immigrants enrolled between 1999 and 2009 in the Campania region.

A meta-analysis published in 2015 by Greenaway and co-workers [53] included 50 studies reporting the HCV seroprevalence in immigrants born in low-income areas and living in developed countries with a low/intermediate HCV prevalence. Out of the 38 635 subjects investigated, 1.9% were anti-HCV-positive (0.63% in the pediatric and 2.34% in the adult population); this rate was 0.77% in immigrants from Latin America and the Caribbean, 4.39% in those from sub-Saharan Africa and 4.76% in those from southern Asia. HCV RNA was determined only in six of the 50 studies considered, accounting for 13% of the 38 635 immigrants.

Interesting are also the data from a retrospective database study describing the epidemiology of HCV-infected patients in the Maccabi Healthcare Services, a health maintenance organization in Israel. The highest prevalence was found among males and in patients aged 35–54 years; two thirds of HCV-infected patients were immigrants from the former Soviet Union [54].

All of these studies highlight the need for systematic screening directed to immigrants from low-income endemic areas, who, despite a high prevalence of cases with HCV chronic infection, are often unaware of their clinical condition [42], as sometimes occurs even among the native population [41].

## Management of HCV chronic infection and associated diseases in immigrant populations

Different types of barriers hinder the access of immigrants to optimal health care services, such as patient-physician communication, language problems, beliefs, traditional medicine, ethnic disparities and inadequacies arising from socioeconomic problems including lack of family support [55–58]. Villagran et al. observed that beliefs about complementary and alternative medicine are negatively related to medical adherence in Mexican immigrants in Texas [59]. Immigrants usually lack adequate knowledge on HCV infection, correlated diseases and treatment [56] and the HCV-infected remain undiagnosed and untreated [57–61]. Left untreated, approximately 20% of individuals with chronic HCV infection progress to liver cirrhosis, which may lead to an end-stage liver disease and HCC development [61, 62]. Immigrants from an endemic area and/or at risk of having acquired HCV, HBV or HIV infection and seen for a consultation at a clinical centre of the National Healthcare System or of a charity organization should be informed of ways to prevent the transmission of infections, on the related clinical events and treatments and on the advantage of a free-of-charge anonymous screening.

### Screening programs

The organization of adequate and functional screening programs for infectious diseases in immigrants is complicated as they cannot easily access National Healthcare Systems; the literature does not contain much research on this topic. Furthermore, in some countries there are no screening programs for immigrants. In the US, the immigration medical examination does not include tests for viral hepatitis [63]. Similarly, universal testing for viral hepatitis is not mandated in Canada, even in patients originating from countries with high prevalence [64]. Instead, the immigration medical screening policies in the EU region are country-specific [55]. Hepatitis screening is currently not required as a condition of entry into the EU region, but some countries have developed programs to screen pregnant mothers from high prevalence regions for HBV infection considering the high risk of maternal-fetal transmission, and then vaccinate their newborns [65, 66].

Our research group screening for HBV, HCV and HIV infection in 2032 undocumented immigrants and low-income refugees was performed in 2012–2013 in southern Italy, involving primary-care outpatient’s clinics in hospitals of the National Healthcare System or of international charity organizations [48, 49, 67–69]. The immigrants seen for a clinical consultation were made aware of the importance of these infections by the physician in care with the help of a cultural mediator and asked to undergo screening for HBV, HCV and HIV infection, offered free of charge and in anonymity. As regards HCV infection, 70 (4.1%) were anti-HCV-positive, all unaware of their serological condition, of whom 31 (44.3%) were HCV-RNA-positive.

### Treatment and management of HCV infected immigrants

Few studies have been published at present on the care and treatment outcome of HCV-positive immigrants treated with oral interferon-free DAA therapies, possibly because of the unavailability of drugs in some countries and of the numerous barriers impeding successful treatment, such as a scanty possibility of screening, poor knowledge of one’s clinical condition, fear of side effects of treatment, late submission to a clinical center authorized for the free administration of drugs and poor adherence to treatment follow-up. [70–78].

In our experience, the 31 HCV-RNA-positive migrants were included in a clinical therapeutic follow-up at a third-level unit of infectious diseases, assisted by a cultural mediator [49]. At that time, following the instructions of the Italian Healthcare Authorities, an oral interferon-free DAA regimen was reserved for cirrhotic patients, whereas those at high risk of developing cirrhosis were to be treated with an Interferon-based treatment and those at low risk of progression left untreated in a waiting list for future interferon-free treatment, once licensed. Of the 31 HCV-RNA-positive immigrants, two had liver cirrhosis, received an interferon-free DAA regimen (one with sofosbuvir + ledipasvir + ribavirin for 3 months and the other with sofosbuvir + daclatasvir + ribavirin for 6 months) and achieved an SVR; 18 had chronic hepatitis, six of whom at high risk of progression to cirrhosis received interferon-based therapy, with SVR in four, and 12 at a low risk of progression were put on a waiting list for future interferon-free treatment [49].

Similarly, in Israel, where the HCV population is comprised of a majority of immigrants, rapid access to DAAs was provided and high SVR rates were achieved [79, 80].

To this regard, it should be stressed that since the DAA regimen may be different for different HCV genotypes that may vary in different countries, it is important to identify the HCV genotype in anti-HCV-positive immigrants. Table 3 shows the distribution of HCV genotypes according to the geographical areas (Table 3). Depending on the distribution of HCV genotypes in the host and origin countries, the migratory flows may modify the genotypic distribution of HCV in Western Europe [37, 81], especially in the case of prolonged migratory flows, as currently occurs from Africa to Europe [82, 83]. In north Africa HCV-genotype 1 and 2 predominate, except for Libya and Egypt where genotype 4 is the most commonly detected [84–86]. The distribution of HCV genotypes in sub-Saharan African is varied. In good agreement with previous studies [84–89], immigrants from West Africa predominantly carry genotypes 2, 3, and 1, those from the horn of Africa genotypes 4, 2, 5 and 1 and those from Chad genotypes 4, 5, and 3.

**Table 3:** Distribution of HCV genotype in African and Asian geographical areas

Country	Prevalent HCV genotype	References
North Africa	1 and 2	83–87
Libya and Egypt	4	84–87
Central Africa	4, 5, and 3	88–89
West Africa	2, 3, and 1	90
Horn of Africa	4, 2, 5, and 1	85
South Asia	3	91–93
Thailand	1	92
Vietnam and Japan	1	93
China	1b, 2a, 3 and 6	94

HCV genotype 3 predominates in southern Asian [90–94], except Thailand [91], Vietnam and Japan [92] where genotype 1 is prevalent. HCV subtypes 1b and 2a predominated in China, but more recently genotype 3 and 6 have spread from southern and western China all over the country [90–93].

Moreover, in immigrants the therapeutic management is complicated. In fact, when the clinical staging of chronic hepatitis is carried out and treatment is started, a whole series of problems related to clinical, biochemical and virological monitoring begin.

For these reasons, treatment of HCV-RNA-positive subjects should be monitored by physicians with extensive professional experience since immigrants are 2–5 times more likely to die from liver disease [60] than the autochthonous individuals and 2–3 fold more likely to develop HCC [61, 62], with HCC-related mortality higher in the immigrant than in the native-born cohorts [59].

To remove such barriers each HCV-RNA-positive subject should receive a culturally appropriate education program, including the use of brochures in the immigrant’s language and the help of a cultural mediator. For example, the program performed in Ontario, the province in Canada with the largest immigrant population, underlines the short duration, good tolerability and high efficacy of the DAAs. In fact, a similar SVR was observed in immigrant and native DAAs recipients (93% in Canadian-born and 98% in immigrants) [95, 96].

## Conclusions

The burden of chronic liver disease in developed countries is now being borne by immigrants who may not have good access to healthcare systems, so despite the extremely high cost of the new DAAs, achieving eradication of HCV infection in immigrants has economic implications and a public health impact. This is also mandatory to obtain the ambitious WHO target of “eradicating” HCV as a public health problem by 2030.

Routine screening of migrants for viral hepatitis is not being performed in the majority of host nations and we think that the Healthcare Authorities should support dedicated screening programs for immigrants, performed with the help of cultural mediators to break down the barriers limiting access to treatments. This may be obtained by using educational brochures and video materials written in the language of the country of origin of the immigrants, explaining in a simple way how HCV is transmitted, how such transmission could be prevented, the clinical progression in time of the associated illness to liver cirrhosis and HCC and how this progression could be prevented by DAA treatments. Once on treatment, adherence to DAA therapy should be accompanied by frequent controls and by the support of a cultural mediator and of skilled physicians. Patients should also be investigated for co-morbidities (HBV coinfection, HIV coinfection, diabetes, mellitus and arterial hypertension) and treated if necessary. For patients with cirrhosis, close monitoring of complications and of HCC development is mandatory even after an SVR has been achieved, including ultrasound examination and liver biochemistry, according to current international guidelines; in addition, monitoring of portal hypertension and other complications is required for cirrhotic patients.

Attention must also be extended to the family nucleus and to the lifestyle of the immigrants with HCV infection, to be of help in the resolution of health and bureaucratic problems; in doing so the role of the cultural mediator is essential.

## Additional file

Additional file 1: Multilingual abstracts in the five official working languages of the United Nations. (PDF 612 kb)

## Abbreviations

DAA: Directly acting antiviral agents
HBV: Hepatitis B virus
HCC: Hepatocellular carcinoma
HCV: Hepatitis C virus
HIV: Human immunodeficiency virus
NS3: Non-structural 3 region
NS5A: Non-structural protein 5A
NS5B: Non-structural protein 5B
peg-IFN: Pegylated-interferon-α
RBV: Ribavirin
SVR: Sustained virological response
