# Supplementary material for: Epidemiology and management of hepatitis C virus infections in immigrant populations
Source: Infect Dis Poverty. 2019 Mar 15;8:17. doi: 10.1186/s40249-019-0528-6 (PMC6419370; doi:10.1186/s40249-019-0528-6)
Supplement: Supplementary file 1 — Multilingual abstracts in the five official working languages of the United Nations. (PDF 612 kb) [file 40249_2019_528_MOESM1_ESM.pdf]

Translation of the abstract into the five official working languages of the United Nations

علم الأوبئة وإدارة عدوى فيروس الالتهاب الكبدي الوبائي في السكان المهاجرين

Nicola Coppola, Loredana Alessio, Lorenzo Onorato, Caterina Sagnelli, Margherita Macera, Evangelista Sagnelli and Mariantonietta Pisaturo

نُبذة

لمحة عامة : في الوقت الحاضر ، هناك تدفق مستمر للمهاجرين من جنوب العالم إلى بلدان الشمال الغربي.غالبا ما ينشأ المهاجرين من مناطق عالية الانتشار بالتهاب الكبد الفيروسي ويشكلون تحديا لنظم الرعاية الصحية في الدول المضيفة. تهدف هذه الدراسة إلى تقييم الانتشار والخصائص الفيروسية والسريريةمن فيروس الالتهاب الكبدي الوبائي عدوى (فيروس الالتهاب الكبدي الوبائي) في المهاجرين والاستراتيجيات لتحديد ورعاية المهاجرين المصابين بفيروس الالتهاب الكبدي الوبائي.

الموضوع الرئيسي:أجرينا بحثًا إلكترونيًا في العديد من قواعد البيانات الطبية الحيوية ، بما في ذلك PubMed و Google Scholar و Scopus و Web of Science باستخدام تركيبات مختلفة من الكلمات الرئيسية: "عدوى فيروس الألتهاب الكبدي الوبائي؛ التهاب الكبد الوبائي المزمن ، والمهاجرين ؛ البلدان ذات الدخل المنخفض". لقد أدرجنا دراسات مكتوبة باللغة الإنجليزية تشير إلى البيانات الوبائية لعدوي فيروس الألتهاب الكبدي الوبائي لدى السكان المهاجرين ، والدراسات التي قيمت العرض السريري ، والإدارة والعلاج السريري مع عامل مضاد للفيروسات يعمل بشكل مباشر في المهاجرين ،

إن عدوي فيروس الألتهاب الكبدي الوبائي منتشرة بشكل غير متساوي في بلدان مختلفة ، مع انتشار عالمي في عموم السكان تتراوح بين 0.5٪ إلى 6.5٪. في البلدان الغربية وأستراليا يتراوح هذا المعدل من 0.5 إلى 1.5٪ ، ويصل إلى 2.3٪ في دول جنوب شرق آسيا وشرق البحر الأبيض المتوسط ، 3.2٪ في الصين ، 0.9٪ في الهند ، 2.2٪ في إندونيسيا و 6.5٪ في باكستان. في أفريقيا جنوب الصحراء الكبرى ، يتراوح معدل انتشار عدوي فيروس الألتهاب الكبدي الوبائي من 4٪ إلى 9٪. المهاجرون واللاجئون القادمون من بلدان مستوطنة من فيروس ال المتوسط / العالي إلى المناطق الأقل أو غير الموبوءة هم أكثر عرضة لزيادة خطر الإصابة بفيروس الألتهاب الكبدي الوبائي بسبب التعرض له في بلدانهم الأصلية. وبسبب ارتفاع معدل الإصابة بفيروس الألتهاب الكبدي الوبائي (HCV) بين السكان المهاجرين والفعالية العالية للعلاج المباشر بعامل مضاد للفيروسات ، يمكن إجراء حملة للقضاء على العدوى في هذا الوضع.

الاستنتاج: يجب أن تدعم سلطات الرعاية الصحية برامج الفحص للمهاجرين ، التي تتم بمساعدة الوسطاء الثقافيين ، بما في ذلك الجوانب التعليمية لكسر الحواجز التي تحد من الوصول إلى العلاجات ، والتي تحصل على إزالة بفيروس الألتهاب الكبدي C في 95 ٪ من الحالات وكثيرا ما تمنع تطور تليف الكبد و سرطان الكبد.

Translated from English version into Arabic by Mohamed Shawkat, proofread by Aalya Al-Beeshi, through

移民人群中丙型肝炎病毒感染的流行病学和管理

Nicola Coppola, Loredana Alessio, Lorenzo Onorato, Caterina Sagnelli, Margherita Macera, Evangelista Sagnelli and Mariantonietta Pisaturo

摘要

引言：目前移民不断从世界南部国家涌入西北部国家。移民通常来自病毒性肝炎高发地区，并对移入国的医疗保健系统构成挑战。本研究的目的是评估移民感染丙型肝炎病毒（HCV）的流行程度、病毒学和临床特征，并且制定用于诊断和管理感染 HCV 移民的策略。

正文：我们使用 PubMed，Google Scholar，Scopus 和 Web of Science 等生物医学数据库进行文献检索。采用不同的关键词组合：HCV 感染；慢性丙型肝炎；移民；低收入国家。纳入的研究为以英文撰写的关于移民人群 HCV 感染的流行病学，这些研究评估临床表现、临床管理和抗病毒药物治疗等。
不同国家的 HCV 感染分布不均，全球患病率为 0.5-6.5％。在西方国家和澳大利亚，患病率在 0.5％至 1.5％之间，在东南亚和东地中海地区升至 2.3％，中国为 3.2％，印度为 0.9％，印度尼西亚为 2.2％，巴基斯坦为 6.5％。在撒哈拉以南非洲地区，HCV 感染的患病率为 4-9％。从中/高度 HCV 流行国家进入低或非流行地区的移民和难民，更有可能因其在原籍国的 HCV 暴露而增加感染 HCV 的风险。由于 HCV 在移民人群中的高度流行以及抗病毒药物治疗的高效性，可以采取措施来消除这种情况下的感染。
结论：医疗机构应支持在文化调解员帮助下进行的移民筛查计划，并利用健康教育来打破限制治疗的障碍。该方法可在 95％的病例中清除 HCV，并且可阻止该病继续发展为肝硬化和肝癌。

Translated from English version into Chinese by Cong-Shan Liu, edited by Pin Yang

CHINESE CENTER FOR DISEASE CONTROL AND PREVENTION
NATIONAL INSTITUTE OF PARASITIC DISEASES

Épidémiologie et prise en charge des infections par le virus de l’hépatite C dans les populations migrantes

Nicola Coppola, Loredana Alessio, Lorenzo Onorato, Caterina Sagnelli, Margherita Macera, Evangelista Sagnelli et Mariantonietta Pisaturo

Résumé

Contexte: On observe actuellement dans le monde un flux continu de migrants des pays du Sud vers les pays du Nord-Ouest.Les migrants viennent souvent de régions où la prévalence de l’hépatite virale est élevée, ce qui représente un défi pour les systèmes de santé des pays hôtes. L’objectif de cette étude est d’évaluer la prévalence et les caractéristiques virologiques et cliniques de l’infection par le virus de l’hépatite C (VHC) chez les migrants et les stratégies d’identification et de prise en charge des migrants infectés par le VHC.
Discussion: Nous avons mené une recherche documentaire électronique dans plusieurs bases de données biomédicales, notamment PubMed, Google Scholar, Scopus et Web of Science, avec différentes combinaisons de mots-clés, à savoir «HCV infection», «chronic hepatitis C, immigrants», «low-income countries» (infection par le VHC, C chronique et immigrants, pays à faibles revenus). Nous avons inclus des études rédigées en anglais et qui fournissaient des données épidémiologiques sur l’infection par le VHC dans des populations migrantes, des études évaluant la présentation clinique, la prise en charge clinique et les traitements avec un agent antiviral à action directe (AAD) parmi des immigrants.
L’infection par le VHC est inégalement répartie entre les différents pays, avec une prévalence mondiale dans la population générale qui va de 0,5% à 6,5%. Dans les pays occidentaux et en Australie, la prévalence est de 0,5% à 1,5%; elle atteint 2,3% dans les pays d’Asie du Sud-Est et de régions de la Méditerranée orientale, 3,2% en Chine, 0,9% en Inde, 2,2% en Indonésie et 6,5% au Pakistan. En Afrique subsaharienne, la prévalence de l’infection par le VHC varie de 4% à 9%. Les immigrants et réfugiés venant de pays où le VHC est endémique avec une prévalence moyenne à élevée et arrivant dans des régions où l’endémie est faible ou l’hépatite B non endémique présentent plus souvent un risque accru d’infection par le VHC en raison de leur exposition au virus dans leur pays d’origine. En raison de l’endémicité élevée du VHC dans les populations migrantes et de la grande efficacité du traitement antiviral à action directe, une campagne pourrait être entreprise pour éradiquer l’infection dans ce contexte.

Conclusions: Il serait utile que les autorités sanitaires soutiennent des programmes de dépistage auprès des immigrants, avec l'aide de médiateurs culturels et en incluant une dimension éducative pour dépasser les obstacles limitant l'accès aux traitements, sachant que ces traitements permettent d'éliminer le VHC dans 95% des cas et empêchent souvent le développement d'une cirrhose du foie et d'un carcinome hépatocellulaire.

Translated from English version into French by Ingrid Vieira Mary, proofread by Suzanne Assenat, through

TRANSLATORS WITHOUT BORDERS

Эпидемиология и лечение инфекций вируса гепатита С среди иммигрантов

Никола Коппола, Лоредана Алессио, Лоренцо Онорато, Катерина Сагнелли, Маргерита Масера, Евангелиста Сагнелли и Мариантониетта Писатуро

Аннотация

Общие сведения: В настоящее время из южной части земного шара в северо-западные страны идет непрерывный поток иммигрантов. Зачастую иммигранты приезжают из регионов с высокими показателями распространенности вирусного гепатита и представляют угрозу для систем здравоохранения принимающих государств. Цель настоящего исследования – проанализировать уровни распространенности и вирусологические и клинические характеристики инфекции вируса гепатита С (ВГС) среди иммигрантов, а также стратегии выявления и лечения иммигрантов, инфицированных ВГС.

Основная часть: Мы осуществили поиск по нескольким электронным базам биомедицинской литературы, включая PubMed, Google Scholar, Scopus, Web of Science, используя различные комбинации ключевых слов: «инфекция ВГС; хронический гепатит С; иммигранты; страны с низким уровнем дохода». Мы включили в поиск исследования на английском языке, содержащие эпидемиологические данные об инфицировании ВГС среди иммигрантов, а также исследования, посвященные клинической картине и лечению инфицированных иммигрантов противовирусными препаратами прямого действия.

Уровни распространенности инфекции ВГС в разных странах варьируются и в среднем составляют от 0,5% до 6,5%. В западных странах и Австралии этот показатель составляет от 0,5% до 1,5%; в странах Юго-Восточной Азии и Восточного Средиземноморья он достигает 2,3%, в Китае – 3,2%, в Индии – 0,9%, в Индонезии – 2,2%, в Пакистане – 6,5%; в странах Африки к югу от Сахары уровень распространенности инфекции ВГС варьируется от 4% до 9%. Иммигранты и беженцы, прибывающие из стран с наиболее высокими показателями распространенности ВГС в страны с низкими показателями, представляют повышенный риск заражения, поскольку сами были подвержены такому риску в стране происхождения. Поскольку процент иммигрантов, инфицированных ВГС, высок, а также с учетом высокой эффективности противовирусных препаратов прямого действия, рекомендуется организовать кампанию по борьбе с ВГС с учетом этих обстоятельств.

Выводы: Государственные органы в сфере здравоохранения должны поддерживать программы медицинского обследования иммигрантов, реализуемые при помощи культурных посредников и включающие образовательные аспекты, направленные на устранение препятствий, ограничивающих доступ к препаратам, обеспечивающим

излечение в 95% случаев и зачастую предотвращающим развитие цирроза печени и гепатоцеллюлярной карциномы.

Translated from English version into Russian by Daria Afanasyeva, proofread by Michael Orlov, through

Epidemiología y tratamiento de las infecciones por el virus de la hepatitis C en poblaciones inmigrantes

Nicola Coppola, Loredana Alessio, Lorenzo Onorato, Caterina Sagnelli, Margherita Macera, Evangelista Sagnelli y Mariantonietta Pisaturo

Resumen

Contexto: en la actualidad, hay un flujo continuo de inmigrantes del sur del mundo hacia los países del noroeste. A menudo, los inmigrantes proceden de zonas donde hay una alta prevalencia de hepatitis viral y suponen un reto para los sistemas sanitarios de los países de acogida. El objetivo de este estudio es evaluar la prevalencia y las características virológicas y clínicas de la infección por el virus de la hepatitis C (VHC) en inmigrantes y las estrategias para identificar y atender a los inmigrantes infectados con el VHC.
Texto principal: realizamos una búsqueda de literatura electrónica en varias bases de datos biomédicas, incluidas PubMed, Google Scholar, Scopus y Web of Science, utilizando diferentes combinaciones de palabras clave: «infección con VHC, hepatitis C crónica, inmigrantes, países con bajos ingresos». Se incluyeron estudios escritos en inglés que indicaban los datos epidemiológicos de la infección con VHC en la población inmigrante, estudios que evaluaban la presentación clínica, la gestión clínica y el tratamiento de inmigrantes con agentes antivirales de acción directa.
La infección con VHC está distribuida de manera desigual en los diferentes países, con una prevalencia mundial en la población general que va del 0,5% al 6,5%. En los países occidentales y en Australia esta tasa va del 0,5 al 1,5% y alcanza el 2,3% en países del sudeste asiático y en la región este del Mediterráneo, un 3,2% en China, un 0,9% en India, un 2,2% en Indonesia y un 6,5% en Pakistán; en la zona subsahariana de África la prevalencia del VHC varía del 4% al 9%. Los inmigrantes y refugiados de países con un nivel endémico medio/alto del VHC en áreas menos endémicas o no endémicas tienen más probabilidades de tener un mayor riesgo de infección con VHC debido a la exposición al VHC en sus países de origen. Debido al carácter endémico elevado del VHC en las poblaciones inmigrantes y a la alta eficacia de la terapia con agentes antivirales de acción directa, podría emprenderse una campaña para erradicar la infección en este entorno.
Conclusiones: las autoridades sanitarias deben apoyar los programas de cribado para inmigrantes, realizados con la ayuda de mediadores culturales e incluyendo aspectos educativos para romper las barreras que limitan el acceso a los tratamientos, que en el 95% de los casos consiguen la eliminación del VHC y que con frecuencia evitan el desarrollo de la cirrosis hepática y del carcinoma hepatocelular.

Translated from English version into Spanish by Lia Sarra Felip, proofread by Mayra León, through
